# First-Principles Insight into Pd-Doped C_3_N Monolayer as a Promising Scavenger for NO, NO_2_ and SO_2_

**DOI:** 10.3390/nano11051267

**Published:** 2021-05-12

**Authors:** Ruochen Peng, Qu Zhou, Wen Zeng

**Affiliations:** 1 College of Engineering and Technology, Southwest University, Chongqing 400715, China; ruochenpeng1207@163.com; 2 College of Materials Science and Engineering, Chongqing University, Chongqing 400044, China

**Keywords:** Pd-C_3_N monolayer, first-principles calculation, toxic gas, adsorption

## Abstract

The adsorption and sensing behavior of three typical industrial toxic gases NO, NO_2_ and SO_2_ by the Pd modified C_3_N monolayer were studied in this work on the basic first principles theory. Meanwhile, the feasibility of using the Pd doped C_3_N monolayer (Pd-C_3_N) as a sensor and adsorbent for industrial toxic gases was discussed. First, the binding energies of two doping systems were compared when Pd was doped in the N-vacancy and C-vacancy sites of C_3_N to choose the more stable doping structure. The result shows that the doping system is more stable when Pd is doped in the N-vacancy site. Then, on the basis of the more stable doping model, the adsorption process of NO, NO_2_ and SO_2_ by the Pd-C_3_N monolayer was simulated. Observing the three gases adsorption systems, it can be found that the gas molecules are all deformed, the adsorption energy (*E_ad_*) and charge transfer (Q_T_) of three adsorption systems are relatively large, especially in the NO_2_ adsorption system. This result suggests that the adsorption of the three gases on Pd-C_3_N belongs to chemisorption. The above conclusions can be further confirmed by subsequent deformable charge density (DCD) and density of state (DOS) analysis. Besides, through analyzing the band structure, the change in electrical conductivity of Pd-C_3_N after gas adsorption was studied, and the sensing mechanism of the resistive Pd-C_3_N toxic gas sensor was obtained. The favorable adsorption properties and sensing mechanism indicate that the toxic gas sensor and adsorbent prepared by Pd-C_3_N have great application potential. Our work may provide some guidance for the application of a new resistive sensor and gas adsorbent Pd-C_3_N in the field of toxic gas monitoring and adsorption.

## 1. Introduction

Nowadays, with the progress of the economy and the acceleration of industrialization, the problem of industrial waste gases is becoming more and more serious. The industrial production process produces a large number of industrial waste gases, and these waste gases’ emission into the air will spread with the flow of the atmosphere, causing great harm to the environment and threatening the physical and mental health of the people [1,2]. NO, NO_2_ and SO_2_ are several typical toxic industrial waste gases. Hence, finding an effective method of detecting and adsorbing these toxic gases is of great significance to protect our living environment.

Since the typical two-dimensional (2D) material graphene was successfully synthesized, 2D nanomaterial, such as transition metal dihalogen compounds [3,4,5], silicene [6,7], germanene [8,9], and stannene [10,11], have attracted extensive attention in academia. The excellent properties of these 2D materials make them have broad application prospects in many areas [12,13,14,15]. Whereas, the zero-band gap characteristic of graphene limits its application in the nanoelectronics field [16,17]. Thus, researchers began to explore novel two-dimensional graphene-like materials, such as metal nitrides and carbides [18], III-V nitrides [19,20,21], etc. Among these new graphene-like materials, a planar honeycomb C_3_N monolayer which can be thought of as a 2 × 2 graphene supercell substituted by two N atoms is emerging [22,23]. C_3_N is a semiconductor, and one of its characteristics compared to graphene is its indirect band gap. Owing to the substitution of N atoms, compared with grapheme, the C_3_N monolayer has higher chemical activity and carrier mobility as well as better structural stability [24,25]. Therefore, C_3_N may have great application potential in gas sensing and adsorption fields [26,27]. Previous studies have shown that intrinsic C_3_N is inert to many toxic gases [28]. The adsorption capacity of nanomaterial with transition metal (TM) doping on gas molecules can be significantly improved due to significant electron hybridization between the TM atom and gas molecule [29,30,31]. Zhu et al. [32] found that the InN monolayer doped with Pd has a good application prospect in detecting and removing toxic gases CO and NO. Ma et al. [33] found that the Au, Pt, Pd and Ni modified MoS_2_ monolayers have good sensing performance for CO and NO gases. Therefore, the doping of Pd may enhance the adsorption capacity of C_3_N to toxic gases NO, NO_2_ and SO_2_. However, so far, few research has been executed in the adsorption properties of the TM doped C_3_N (TM-C_3_N) monolayer for toxic gases such as NO, NO_2_ and SO_2_.

Based on first principles, the doping behavior of Pd on C_3_N and the adsorption properties of Pd-C_3_N for NO, NO_2_ and SO_2_ were studied in this study. Further, to study the adsorption properties and sensing mechanism of Pd-C_3_N for three kinds of toxic gases, the *E_ad_*, Q_T_, DCD, DOS and band structure were analyzed. The results show that NO, NO_2_ and SO_2_ can be stably adsorbed by Pd-C_3_N, which can be recognized as chemisorption. The adsorption properties and sensing mechanism of Pd-C_3_N for toxic gases obtained in this work provide a theoretical basis for further study of the toxic gas resistive sensor and adsorbent prepared by Pd-C_3_N.

## 2. Computation Methods

All the theoretical calculations on the basic density functional theory (DFT) in this paper were carried out in the dispersion-corrected DMol^3^ package [34,35]. The exchange-correlation between electrons was handled by the Perdew–Burke–Ernzerhof (PBE) function under the generalized gradient approximation (GGA) to better describe the non-uniform electron density of the system which was closer to the experimental situation [36,37,38]. The DFT-D method, which was customized by Grimme, was used to understand van der Waals force and long-range interactions better [39]. We used DFT semi-core pseudopotential (DSSP) to handle the effects of core electron relativity and chose double numerical plus polarization (DNP) to calculate the density function of each model [40,41,42]. In terms of the setup of Monkhorst-Pack k-point mesh, 7 × 7 × 1 was set for geometric optimization and 10 × 10 × 1 for the calculation of static electronic structure [43]. The energy tolerance accuracy, maximum force, and displacement were severally set as 10^−5^ Ha, 0.002 Ha/Å and 0.005 Å [44].

A 2 × 2 × 1 C_3_N supercell with 28 C atoms and 13 N atoms was built. In order to prevent the adjacent layers from interacting with each other, the vacuum region was set to 15 Å [45]. The lattice constant of the fully optimized C_3_N monolayer was calculated as 4.92 Å, which was basically consistent with the previous report (4.9 Å [46]). The Hirshfeld method was adopted to study the electronic behavior of atoms and molecules [47]. Meanwhile, charge transfer (Q_T_) is defined to describe the electronic behavior of Pd doping and gas adsorption systems. A positive Q_T_ value means the analyte acts as an electron donator, on the contrary, a negative Q_T_ value means that the analyte acts as an electron acceptor [48].

## 3. Results and Discussions

### 3.1. Isolated Gas Molecules and Pd-C_3_N Monolayer

**Figure 1:**
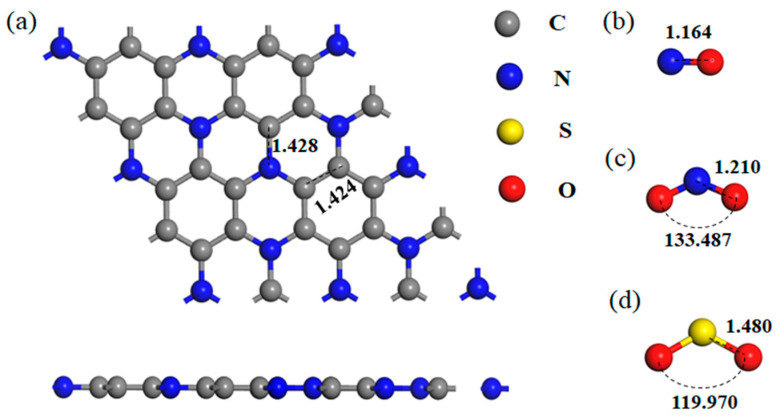
Optimized configuration of (**a**) C_3_N monolayer, (**b**) NO, (**c**) NO_2_ and (**d**) SO_2_.

Figure 1 displays the optimized structural models of the intrinsic C_3_N monolayer and three gas molecules NO, NO_2_ and SO_2_. At the same time, Table 1 lists the geometrical parameters of the optimized three kinds of gas molecular configurations and Table 2 lists the single atom charges of gas molecules in the gas phase.

**Figure 2:**
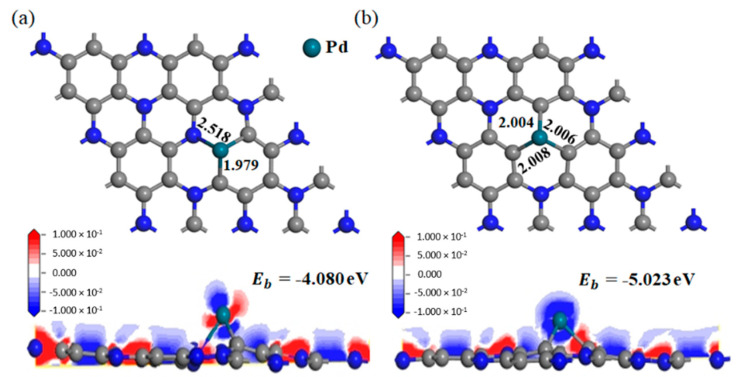
Top view of the optimized structure, side view of DCD (**a**) Pd doped in VC-C_3_N, (**b**) Pd doped in VN-C_3_N.

According to previous report, the metal atom can be stably adsorbed by the C_3_N monolayer with one C atom or one N atom deficiency (simplified as VC-C_3_N and VN-C_3_N below) due to the strong electrostatic attraction where electronic localization occurs [49]. Therefore, to obtain the most stable doping structure, priority was given to the Pd atom doping at the C-vacancy or N-vacancy site on the C_3_N monolayer. The two optimized doping configurations are shown in Figure 2. When Pd is doped at the C-vacancy site, the length of Pd-C and Pd-N is significantly different, which are 1.979 and 2.518 Å, respectively. However, when Pd is doped at the N-vacancy site, the three Pd-C bonds have basically the same length, 2.004, 2.006 and 2.008 Å, respectively. This result shows that the doping system with Pd doping at the N-vacancy site has better central symmetry. In this paper, the stability of the doping system is evaluated by binding energy (*E_b_*), and the calculation formula is as follows:

(1) $$E_{b}{=\;E}_{{Pd-C}_{3}N}-E_{{\mathrm{vacancy}-C}_{3}N}-E_{Pd}$$

In the above formula, $E_{{Pd-C}_{3}N}$ denotes the energy of the Pd-doped system, and $E_{{\mathrm{vacancy}-C}_{3}N}$ and $E_{Pd}$ denote the energy of the defective C_3_N and Pd atom, respectively. The binding energies of Pd doping at the C-vacancy site and N-vacancy site are −4.080 and −5.023 eV, respectively. This result indicates that Pd tends to be doped at the N-vacancy site, because the doping system at this time is more stable.

**Figure 3:**
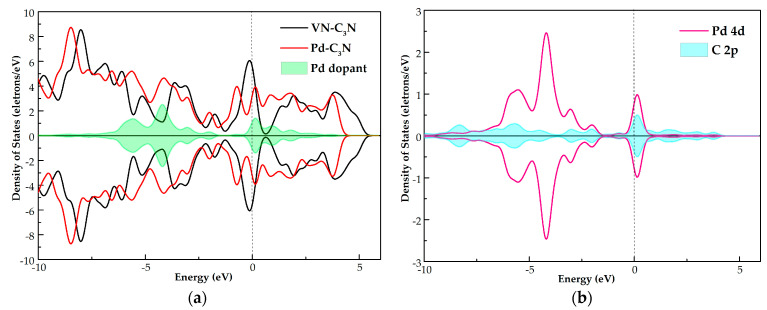
(**a**) TDOS of the VN-C_3_N monolayer, Pd-C_3_N monolayer, (**b**) PDOS of the Pd-C_3_N monolayer, the dotted line indicates the Fermi energy.

To further study the electronic behavior of Pd-C_3_N, we calculated DCD and DOS, as displayed in Figure 2 and Figure 3, respectively. In the DCD of Figure 2, the areas with increased charge density are shown in red, while the areas with decreased charge density are shown in blue. As shown in Figure 2b, in the doping system where Pd doped is in the N-vacancy site, the charge density around Pd and C atoms decreases, while the charge density around N atoms increases. This result implies the electron-losing property of the Pd atom. In other words, the Pd atom transfers electrons to the VN-C_3_N monolayer. Meanwhile, the electron density between the Pd atom and C atom is very high, which suggests that a stable chemical bond in Pd-C is formed, so Pd can be stably adsorbed by VN-C_3_N. As can be seen from the total DOS of Pd-C_3_N, the spin up and spin down curves are highly symmetrical. This phenomenon shows that the doping system is not magnetic. Besides, the doping of Pd induces several impurity states, leading to some new peaks of total DOS after doping in the vicinity of −5.5, −4.0, 0.2, 1.0 and 2.0 eV. New peaks can be observed at the top of the valence band and at the bottom of the conduction band, indicating that Pd doping contributes greatly to the states near the Fermi energy. According to Figure 3b, huge hybridization occurs between Pd 4d orbital and C 2p orbital at multiple energy levels, such as −5.5, −2.7, −2 and 0.2 eV. This phenomenon confirms the previous conclusion that Pd can form a stable chemical bond with C and can be stably adsorbed by VN-C_3_N. Through the analysis of DCD and DOS, it can be concluded that the electronic behavior of VN-C_3_N has a significant change after doping with the Pd atom.

### 3.2. Adsorption Analysis of Pd-C_3_N Monolayer to NO, NO_2_, SO_2_

In order to fully compare various possible configurations of the three gas adsorption systems and find the most stable one for analysis, NO, NO_2_ and SO_2_ were placed in different directions on top of the Pd-C_3_N monolayer. Adsorption energy (*E_ad_*) can describe the energy change of each adsorption structure, so it can be used to assess the stability of the system after adsorption of gas. The calculation formula of *E_ad_* is as below:

(2) $$E_{ad}{=\;E}_{{Pd-C}_{3}N/gas}-E_{{Pd-C}_{3}N}-E_{gas}$$

**Figure 4:**
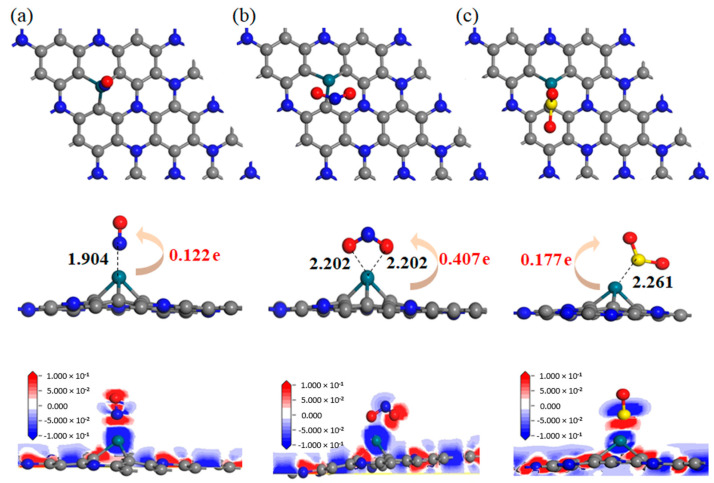
The steadiest adsorption configuration of gas on the Pd-C_3_N monolayer and the DCD of this configuration (**a**) NO; (**b**) NO_2_ and (**c**) SO_2_ adsorption systems.

In the above formula, $E_{{Pd-C}_{3}N/gas}$ and $E_{{Pd-C}_{3}N}$ respectively represent the energy before and after the adsorption of gas by Pd-C_3_N, and $E_{gas}$ represents the energy of the isolated gas molecule. The adsorption energies of the three gas adsorption systems are all negative, suggesting that the gas adsorption process of Pd-C_3_N is accompanied by the release of heat. Choose the structure with the lowest *E_ad_*, that is, the most stable configuration for subsequent works (as displayed in Figure 4). To understand the mechanism of charge transfer better, deformed charge density (DCD) is also described in Figure 4. Meanwhile, Table 3 and Table 4 show the specific characteristic parameters of the three gas adsorption systems.

In the NO adsorption system, the NO molecule is adsorbed on top of the Pd atom and perpendicular to the C_3_N plane. When NO is adsorbed, the N-O bond elongates from 1.164 to 1.188 Å, indicating that the NO molecule has certain activity during the adsorption process. The *E_ad_* of the NO adsorption system is −1.83 eV, so the adsorption of NO by the Pd-C_3_N monolayer can be identified as chemisorption. Meanwhile, according to the DCD in Figure 4a, the charge density near N atoms and O atoms increases. From the molecular point of view, NO has a 0.122 e negative charge, indicating the electron-receiving property of NO. During the interaction with the Pd-C_3_N monolayer, NO obtains 0.122 e from it. According to Figure 4b,c, NO_2_ and SO_2_ tend to be adsorbed on one side of the Pd dopant in the Pd-C_3_N monolayer rather than on the top. In addition, the adsorbed NO_2_ and SO_2_ molecules are negatively charged, which means that they both act as electron acceptors to absorb 0.407 e and 0.177 e from the Pd-C_3_N monolayer, respectively. In the NO_2_ adsorption system, the N-O bond elongates to 1.281 Å compared to the 1.210 Å in the isolated phase. At the same time, the O-N-O bond angle of the NO_2_ molecule in the adsorption system decreases from 133.487° in the gas phase to 111.674°. This significant deformation indicates that NO_2_ has obvious geometric activation during its interaction with the Pd dopant. Besides, the charge density near the N atom in the NO_2_ adsorption system decreases, while the charge density near the O atom increases. In addition, the *E_ad_* of the NO_2_ adsorption system is −2.74 eV, which indicates that NO_2_ has an ideal chemisorption on the surface of Pd-C_3_N, which is supported by large Q_T_ (−0.407 e) and geometric deformation. In the SO_2_ adsorption system, the S-O bond elongates from 1.480 to 1.495 Å, while the O-S-O bond angle decreases from 119.970° to 119.932°, suggesting that SO_2_ is activated when interacting with the surface of Pd-C_3_N. The *E_ad_* of the SO_2_ adsorption system is −1.61 eV, Q_T_ is −0.177 e, which can be used to identify the adsorption as chemisorption.

Previous reports have shown that *E_ad_* of the intrinsic C_3_N monolayer adsorption system for NO, NO_2_ and SO_2_ is −0.248, −0.840 and −0.584 eV, respectively [28]. Comparing to the results in this study, it can be found that the adsorption capacity of Pd-C_3_N for NO, NO_2_ and SO_2_ was significantly higher than that of intrinsic C_3_N. At the same time, the adsorption process of three kinds of gas molecules by the Pd-C_3_N monolayer is accompanied by a relatively large charge transfer, which indicates that the adsorption of gas will lead to the redistribution of electrons in the whole system and change the electronic behavior of Pd-C_3_N. To further explore the electronic behavior of Pd-C_3_N during the adsorption of NO, NO_2_ and SO_2_, DOS is analyzed in the following.

### 3.3. DOS Analysis of NO, NO_2_ and SO_2_ Adsorption Systems

**Figure 5:**
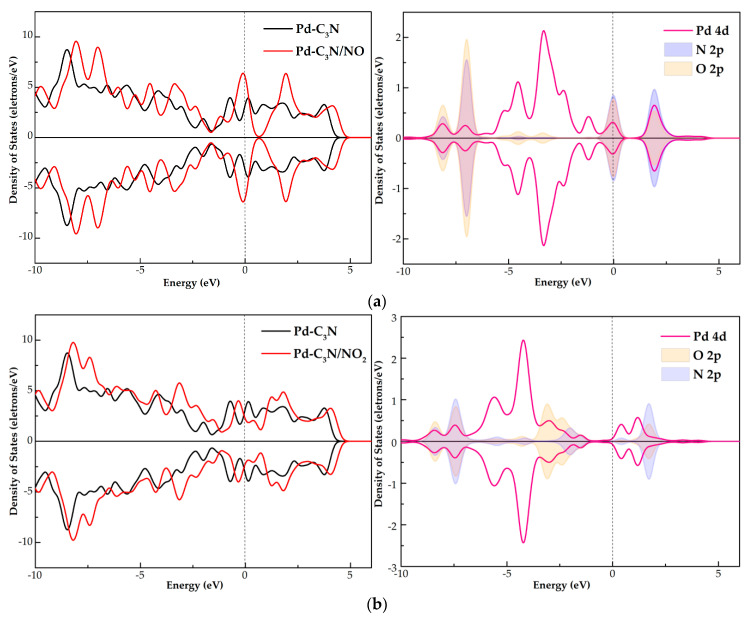

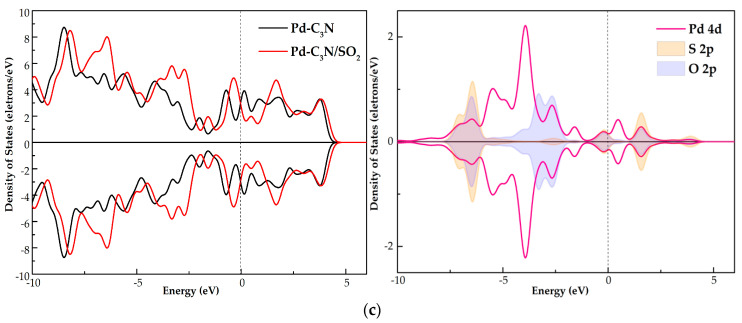
TDOS and PDOS of (**a**) NO, (**b**) NO_2_ and (**c**) SO_2_ adsorption systems, the dotted line indicates the Fermi energy.

DOS is an important parameter for studying the electronic behavior of the interaction between gas and the Pd-C_3_N surface. According to Figure 5, the total DOS (TDOS) of the three adsorption systems shift to the right in different degrees compared with the Pd-C_3_N monolayer, and some new peaks appear nearby the Fermi level. In the TDOS of NO adsorption system, novel peaks appear in the vicinity of −1, −0.1 and 2 eV, while in NO_2_ and SO_2_ adsorption systems, the new peaks appear in the vicinity of −1.5, −0.3 and 0.3 eV. Besides, there are multiple activated states in the gas molecules due to the interaction between it and the surface of the Pd dopant. Then, the orbital hybridization of these activated states with Pd 4d results in new peaks in the TDOS of the three adsorption systems. In particular, the activated states of gas molecules lead to a certain degree of deformation of the states at the top of the conduction band and the bottom of the valence band, which indicates that the adsorption of gas will affect the electronic behavior of Pd-C_3_N.

Atomic DOS (PDOS) is shown in Figure 5. In the NO adsorption system, the N 2p and O 2p orbitals of activated NO have certain hybridization with Pd 4d orbitals at −8, −7, 0 (Fermi level) and 2.2 eV. According to the atomic DOS of the NO_2_ adsorption system, the Pd 4d orbital is strongly hybridized with N 2p and O 2p orbitals around −8, −7.2 and 2 eV, resulting in a relatively large charge transfer between NO_2_ and Pd-C_3_N. In the atomic DOS of SO_2_ adsorption system, S 2p, O 2p and Pd 4d orbital have strong hybridization at energy levels of −6.3, −2.5, −0.2 and 2 eV, indicating that there is a good orbital interaction between SO_2_ and Pd dopant. The strong hybridization between the atomic orbitals of the three gas molecules and Pd 4d orbital again confirms that NO, NO_2_ and SO_2_ can be stably adsorbed by Pd-C_3_N.

In summary, the strong interaction between three gases and Pd-C_3_N during gas adsorption process significantly affects the electronic behavior of Pd-C_3_N.

### 3.4. Band Structure Analysis of NO, NO_2_ and SO_2_ Systems

**Figure 6:**
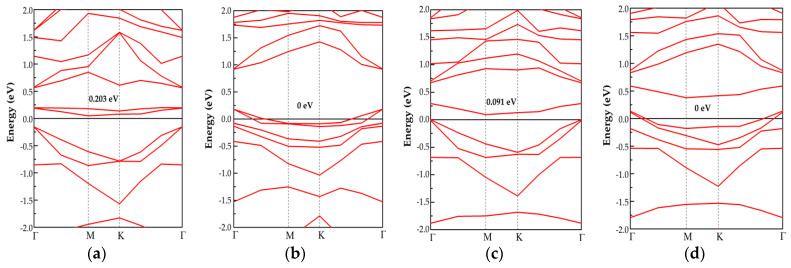
Energy band structure of (**a**) Pd-C_3_N monolayer and (**b**) Pd-C_3_N/NO, (**c**) Pd-C_3_N/NO_2_, (**d**) Pd-C_3_N/SO_2_ systems.

To further study the change in electrical conductivity of Pd-C_3_N after adsorbing gas, we calculated and analyzed the band structure of three adsorption systems (Figure 6). In the band structure, the energy interval with zero energy state density between the conduction band and the valence band is called the band gap [50,51]. The narrower the band gap, the more easily the electron can be excited across the band gap, the higher the conductivity. According to Figure 6a, the band gap of the Pd-C_3_N monolayer is 0.203 eV, which is much narrower than that of C_3_N (0.44 eV [52]). Besides, the band structure of the doping system does not have an impurity state beyond the Fermi level. Thus, C_3_N doped with the Pd atom still has semiconductor property. In the band structure of NO and SO_2_ adsorption systems (Figure 6b,d), the new impurity level surpassing the Fermi energy appears at the top of the valence band, causing a zero band gap for both systems. Therefore, the adsorption of NO and SO_2_ can be deemed to strong p-type doping for Pd-C_3_N [53]. According to Figure 6, the band gap of NO and SO_2_ adsorption system is 0 eV, while that of NO_2_ adsorption system is 0.091 eV. It can be seen that the band gaps of these three adsorption systems are much narrower than that of Pd-C_3_N. This result shows that the conductivity of Pd-C_3_N is observably improved after adsorbing gas, especially after adsorbing NO and SO_2_. Through calculating and analyzing the band structure of the three adsorption systems, it is helpful to further understand the sensing mechanism of the resistive chemical sensor prepared by Pd-C_3_N.

## 4. Conclusions

The adsorption performance and sensing mechanism of the Pd-C_3_N monolayer for three kinds of industrial toxic gases NO, NO_2_ and SO_2_ were explored based on first principles. The DCD, DOS and band structure were considered to study the change in electronic behavior and conductivity of Pd-C_3_N after adsorbing gas. The main conclusions of this study are listed as below:

1. The Pd dopant is more likely to be adsorbed on the N-vacancy site of the C_3_N than the C-vacancy site, because the lower binding energy (*E_b_* = −5.023 eV) of this doping system implies a more stable structure.
2. NO, NO_2_ and SO_2_ can be stably adsorbed by the Pd-C_3_N monolayer and the adsorption can be identified as chemisorption. Besides, the adsorption energy (E_ad_) of the Pd-C_3_N/gas system is much higher than that of the C_3_N/gas system. Among three gas adsorption systems, *E_ad_* and Q_T_ of the NO_2_ system are the largest, which indicates that Pd-C_3_N has the strongest adsorption performance for NO_2_.
3. Through the analysis of DOS, it is found that the gas molecules are activated during the interaction with the Pd dopant surface. The orbital hybridization of these activated states with Pd 4d give rise to new peaks in the TDOS of the three adsorption systems, which influences the electronic behavior of Pd-C_3_N.
4. Through analyzing the band structure, it can be discovered that the band gap of Pd-C_3_N becomes narrower after adsorbing NO, NO_2_ and SO_2_, which significantly improves the conductivity of Pd-C_3_N, especially after adsorbing NO and SO_2_.

To sum up, the calculation in this paper can offer some theoretical basis for the further study of Pd-C_3_N as a resistive sensor and gas adsorbent for the monitoring and adsorption of typical industrial toxic gases in the environment.

## Figures and Tables

**Table 1:** Geometrical parameters of NO, NO_2_ and SO_2_.

Gas	Bond Length (Å)		Bond Angle (°)	
NO	N-O	1.164	-	-
NO_2_	N-O	1.210	O-N-O	133.487
SO_2_	S-O	1.480	O-S-O	119.970

**Table 2:** Single atom charges of gas molecules in the gas phase.

Gas	N	O	S
NO	0.035	−0.035	-
NO_2_	0.375	−0.188	-
SO_2_	-	0.454	−0.227

**Table 3:** The geometrical parameters of three gas adsorption systems.

System	The Length of Bond (Å)		Bond Angle (°)		Adsorption Distance (Å)
Pd-C_3_N + NO	N-O	1.188	-		1.904
	Pd-C	2.046, 2.046, 2.050			
Pd-C_3_N + NO_2_	N-O	1.281	O-N-O	111.674	2.202
	Pd-C	1.978, 1.979, 2.016			
Pd-C_3_N + SO_2_	S-O	1.495	O-S-O	119.932	2.261
	Pd-C	2.046, 2.044, 1.990			

**Table 4:** The characteristic parameters of three gas adsorption systems.

System	Atom	Mulliken Charge (e)	Q_T_ (e)	E_ad_ (eV)
Pd-C_3_N + NO	N	−0.033	−0.122	−1.83
	O	−0.089		
Pd-C_3_N + NO_2_	N	0.270	−0.407	−2.74
	O_1_	−0.338		
	O_2_	−0.339		
Pd-C_3_N + SO_2_	S	0.457	−0.177	−1.61
	O_1_	−0.303		
	O_2_	−0.331		

## Data Availability

The data is available on the request from corresponding author.
